# Synthesis of Bio-Based Poly(Butylene Adipate-*co*-Butylene Itaconate) Copolyesters with Pentaerythritol: A Thermal, Mechanical, Rheological, and Molecular Dynamics Simulation Study

**DOI:** 10.3390/polym12092006

**Published:** 2020-09-03

**Authors:** Chin-Wen Chen, Hsu-I Mao, Zhi-Yu Yang, Kuan-Wei Huang, Hao-Chen Yan, Syang-Peng Rwei

**Affiliations:** Institute of Organic and Polymeric Materials, Research and Development Center of Smart Textile Technology, National Taipei University of Technology, No. 1, Sec. 3, Chung-Hsiao East Road, Taipei 10608, Taiwan; cwchen@ntut.edu.tw (C.-W.C.); eeric0212@hotmail.com (H.-I.M.); andyyang821222@gmail.com (Z.-Y.Y.); wewe85625@gmail.com (K.-W.H.); t106518016@ntut.edu.tw (H.-C.Y.)

**Keywords:** poly(butylene adipate), poly(butylene itaconate), pentaerythritol, slight cross-linking, thermoplastic, copolyester

## Abstract

Bio-based unsaturated poly(butylene adipate-co-butylene itaconate) (PBABI) aliphatic copolyesters were synthesized with pentaerythritol (PE) as a modifier, observing the melting point, crystallization, and glass transition temperatures were decreased from 59.5 to 19.5 °C and 28.2 to −9.1 °C as an increase of itaconate concentration, and T_g_ ranged from −54.6 to −48.1 °C. PBABI copolyesters tend to the amorphous state by the existence of the BI unit above 40 mol%. The yield strength, elongation, and Young’s modulus at different BA/BI ratios were valued in a range of 13.2–13.8 MPa, 575.2–838.5%, and 65.1–83.8 MPa, respectively. Shear-thinning behavior was obtained in all BA/BI ratios of PBABI copolyesters around an angular frequency range of 20–30 rad s^−1^. Furthermore, the thermal and mechanical properties of PBABI copolyesters can be well regulated via controlling the itaconic acid contents and adding the modifier. PBABI copolyesters can be coated on a 3D air mesh polyester fabric to reinforce the mechanical property for replacing traditional plaster applications.

## 1. Introduction

Aliphatic copolyesters can be an eco-friendly, green material, bioresource, and excellent biodegradability material, and have a flexible chain and low melting point to limit their commercial applications due to weak mechanical characteristics [1,2,3,4,5]. Hence, there are many methods to improve their mechanical properties, such as adding nanoparticles, crosslinking agents, chain extenders, etc. A crosslinking agent is an easy way to enrich the mechanical property, but a harder characteristic occurs with the growth of a crosslinking modifier. Furthermore, the slight content crosslinking modifier can be adopted to tune the mechanical property of synthesizing unsaturated copolyesters to obtain a suitable toughness property for its further applications. 

The modifier can be selected with a multi arms end group with –OH and –COOH residues utilized in copolyesters; e.g., benzene-1,3,5-tricarboxylic acid and glycerol with the tri-arm; 2,2-bis(hydroxymethyl) 1,3-propanediol, ethylenediaminetetraacetic acid, and 1,2,4,5-benzenetetracarboxylic acid with the tetra-arm; cyclohexane-1,2,3,4,5,6-hexacarboxylic acid and 1,2,3,4,5,6-cyclohexanehexol with the hexa-arm. A slight content (0.01–0.3 mole%) of multifunctional end groups on aliphatic copolyesters has been explored to form a partial networking architecture, for which the thermal and mechanical properties were accurately regulated, while still maintaining the thermoplastic’s properties and easy processability [6,7,8,9]. For tri-arm modifiers, Chan and coworkers produced the poly(ethylene sebacate-*co*-ethylene adipate) (PESA) with benzene-1,3,5-tricarboxylic acid, observing a lower melting point in the rane of 60–70 °C, a relative higher Young’s modulus in a range of 140–200 MPa, and elongation in 35–75% due to a partial crosslinking network generated [6]. Hsu et al. developed a fully biobased poly(butylene succinate-*co*-propylene succinate) (PBSPS) system with glycerol adjust the thermal-mechanical properties, revealing the PBSPS copolyesters could be changed from elastic to rigid characteristics by increasing the PS unit, proposing that the elongation decreased from 800% to 20% and Young’s modulus ranged from 288.19–58.11 MPa [7]. Furthermore, for tetra-arm modifiers, Chen and colleagues developed unsaturated poly(butylene adipate-*co*-butylene itaconate) (PBABI) copolyester, which copolymerized with ethylenediaminetetraacetic acid (EDTA) [8] and 1,2,4,5-benzenetetracarboxylic acid (BTCA) [9] to adjust the thermal and mechanical properties. Otherwise, the slight crosslinking modifiers were the better choice to form the networking architecture to improve the crystallization rate, mechanical and thermal properties of these kinds of aliphatic copolyesters. Young’s modulus was measured to get to 0.77–128.65 MPa and 32.19–168.45 MPa, and elongation ranged from 71.04–531.76% and 13.78–56.13% in the presence of 0.1 mol% EDTA and BTCA, respectively. As described above, the thermal and mechanical behaviors of aliphatic polyester can be tuned by tri- or tetra-arm crosslinking modifiers due to a partial networking architecture formed. Additionally, another tetra-arm modifier, pentaerythritol (PE), is an organic compound with the formula C(CH_2_OH)_4_ has a tetra-functional –OH, which can be reacted with –COOH to form ester-based copolymers. PE acts as a building block for the synthesis and production of alkyd resins, varnishes, polyvinyl chloride stabilizers, tall oil esters, olefin antioxidants, and many other commercial products. Furthermore, PE has a tetra-functional –OH group, which can be passed in copolyesters to drive crosslinking networking to improve the mechanical property [10,11,12,13,14,15,16,17,18,19,20,21,22,23,24,25,26,27,28,29,30]. Amarasekara et al. synthesized poly(levulinic acid–pentaerythritol), exhibiting a crosslinked structure and can be applied in producing household items, machine parts, medical equipment, packaging, and storage containers [10]. Lu and his coworkers developed poly(butylene succinate-*co*-terephthalate) copolyesters with a small amount of 0.1–0.4 mol% PE to generate a long-chain branched structure, indicating the strength and elongation increased by 4% and 15% in 0.1 mol% PE and the tensile modulus increased and reached a plateau of 132 MPa at PE over 0.4 mol% [17]. Park and colleagues studied branched poly(1,4-butylene carbonate-*co*-terephthalate)s with PE content below 0.3 mol% to avoid gel formation, showing remarkable improved mechanical properties and melt processability as a biodegradable polymer [21]. Ammarah Mahmud et al. established oleic acid-based copolyester with PE to observe a good pour point ranging from −42 to −59 °C and high flash points of 280–300 °C, to improve the lubricity performance [18]. Kricheldorf and Behnken adopted PE to copolymerize with dimethyl sebacate to develop biodegradable and soluble hyperbranched aliphatic polyesters for coating usage. Shim and coworkers synthesized crosslinked poly(ethylene terephthalate-*co*-ethylene glycol) (PET-*co*-PEG) copolymers with PE for shape memory application, exhibiting the highest shape recovery rate is observed composed of 30 mol% PEG-200 and 2.5 mol% PE [14]. Liu et al. synthesized an aliphatic copolyester comprising caprolactone, adipic acid, and 1,6-hexanediol, using PE as the crosslinking agent, suggesting the water absorption behavior and degradation rate increased with increasing PE content [15]. Nagata et al. studied a network aliphatic polyesters based on PE with different lengths of diacid to obtain excellent enzymatic degradability [20]. As mentioned above, PE can react with the diacid group to form aliphatic copolyesters and drive a crosslinking architecture to improve the thermal, mechanical, and degradability properties.

We aim to synthesize the PBABI copolyesters with different BA/BI ratios and a constant PE content of 0.1 mol% to investigate the effect of BI concentration and PE modifier on thermal, mechanical, and rheological properties. We observed that a slight PE ratio in 0.1 mol% can improve the tough performance of PBABI copolyesters and exhibit significantly different mechanical behaviors compared to other 4-arm modifiers of EDTA [8] and BTCA [9] at the same BA/BI ratio and modifier content. In order to understand the relationship among these three modifiers in PBABI copolyesters and their effect on mechanical behavior, we have built a simulation model to discover the difference in mechanical property with three different four-arm crosslinking modifiers of PE, BTCA, and EDTA within PBABI copolyesters via all-atom molecular dynamics simulation techniques.

## 2. Experimental

### 2.1. Materials

Itaconic acid (IA, 99%) and 1,4-butanediol (BDO, 99%) were acquired from the First Chemical Corporation (Taipei, Taiwan), and Adipic acid (AA, 99.8%) was given by Asahi Kasei Corporation (Tokyo, Japan). Pentaerythritol (PE, 99%), 4-Methoxyphenol (99%), titanium butoxide (Ti(OBu)4, 97%), deuterium trifluoroacetic corrosive (d-TFA, 99.5%), phenol (≥99.5%), and tetrachloroethane (≥98%) were provided from Sigma-Aldrich (St. Louis, MO, USA), and Dibutyltin dilaurate (DBTDL, 95%) was taken from Alfa Aesar (Haverhill, MA, USA). All the mentioned chemicals were utilized in bulk polymerization with no purification.

### 2.2. Sample Preparation

**Scheme 1:**
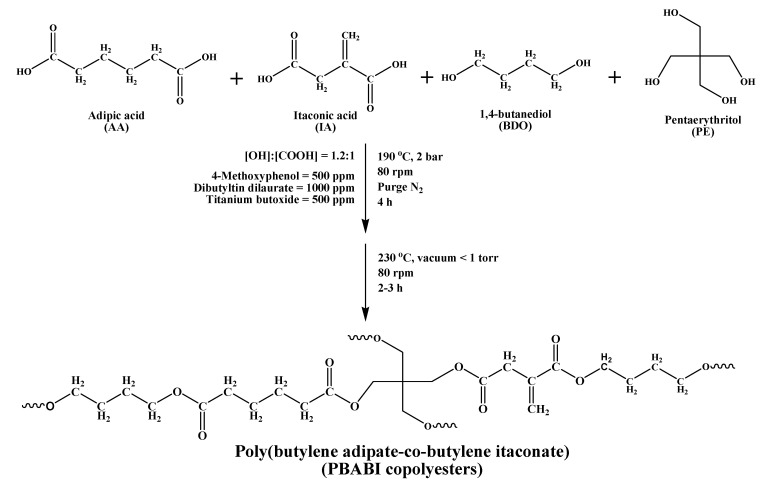
Synthesis route and operation conditions of poly(butylene adipate-co-butylene itaconate) (PBABI) copolyesters.

PBABI copolyesters with PE modifiers were manufactured with via one-pot bulk polymerization techniques. Initially, BDO and PE were esterified with AA and IA, 4-Methoxyphenol as an inhibitor, DBTDL, as well as Ti(OBu)_4_ serving as a catalyst by means of a 2 L steel reactor. PBABI copolyesters represented to as BA/BI = x/y correspond to the molar proportion of butylene adipate (BA)/butylene itaconate (BI) units in the various BA/BI rates of 10/0 to 5/5. The general molar ratio of [OH]/[COOH] was preserved at a molar proportion of 1.2/1, and 4-Methoxyphenol, DBTEL, Ti(OBu)_4_ was included 500, 1000, and 500 ppm, respectively. PE concentration was executed at 0.1 mole% in all BA/BI proportions, and also the synthesizing conditions and strategies, as illustrated in Scheme 1.

### 2.3. Identification of PBABI Copolyesters

The manufactured samples were examined as well as identified through nuclear magnetic resonance (NMR) spectrometer (JEOL ECZ600R 600 MHz, Tokyo, Japan). 100 mg of the manufactured sample was dissolved in d-TFA of 1 mL. The solutions were then transferred into 5 mm NMR tubes. All the evaluations were achieved 128 scans to obtain the raw NMR datum, and then Spinworks software (version 4.2.10) was taken on to check out the NMR figures. Furthermore, Fourier transform infrared spectroscopy (FT-IR) Spectrum One Spectrometer (PerkinElmer, MA, USA) mechanic was made use of to examine the functional group of manufactured samples in attenuated total reflection mode with an average signal of 32 co-added scans at a resolution of 4 cm^−1^, and in a range of 400–4000 cm^−1^. For intrinsic viscosity (IV) measurement, the incorporated samples were dissolved in 1.0 g dL^−1^, a mixing solvent of phenol/tetrachloroethane in 60/40 wt%. All the measurements were taken 5 times to achieve the averaged values through an Ubbelodhe viscometer at 25 ± 0.05 °C. The IV of each sample was determined utilizing the Solomon–Ciuta equation:

(1) $$\left[\eta \right]=\frac{\sqrt{\left[2\left\{\frac{t}{t_{0}}-\ln\left(\frac{t}{t_{0}}\right)-1\right\}\right]}}{C}$$

where *C* is the concentration of the solution; *t* is the flow time of the sample solution, and *t*_0_ is the flow time of the pure solvent.

### 2.4. Thermal Analysis of PBABI Copolyesters

First, thermogravimetric analysis (TGA, Hitachi, STA 7200, Tokyo, Japan) was implemented to observe the curves of weight loss and derivative thermogravimetry (DTG) of the synthesized samples. The synthesized samples were determined in 5–10 mg and heated from 50 to 600 °C at a heating rate of 10 °C min^−1^ under a nitrogen atmosphere at a rate of 50 mL min^−1^. The TGA curve was revealed at 5% weight loss (T_d-5%_), and the DTG curve as a function of temperature was also confirmed. Second, differential scanning calorimetry (DSC, Hitachi High Tech. DSC-7000, Tokyo, Japan) was to review the T_m_, T_c_, ΔH_m_, as well as ΔH_c_ of the manufactured samples with 5–10 mg at a heating rate of 10 °C min^−1^ from room temperature (RT) to 100 °C and held for 5 min to eliminate thermal history. Subsequently, these samples were cooled to −70 °C, then reheated from −70 to 100 °C at the same rate of 10 °C min^−1^.

### 2.5. Mechanical Properties of PBABI Copolyesters

Dynamic mechanical analyzer (DMA, Tech Max DMS 6100, Tokyo, Japan) was performed to estimate the viscoelastic properties for storage modulus (E’), loss modulus (E’’), and loss tangent (tan δ). The manufactured samples were prepared at a size of 40 mm in length, 10 mm in width, and 0.2 mm in thickness. Compression mode with 150 mN at a frequency of 1 Hz was selected, and temperature ranged from −150 to 0 °C at a heating rate of 5 °C min^−1^. In the Tensile test, hot melt compression molding was employed to prepare dumb−bell shaped samples at 80 °C and 3 kg cm^−2^ in 2 min. Then, the measurements were taken at a crosshead speed of 50 mm min^−1^ via Cometech QC-508M2F equipment (Taichung, Taiwan) based on the ASTM d638 Type IV (Length: 33 mm, width: 6 mm, thicken: 3 mm, the gauge length being 50 mm) standard to achieve a stress–strain curve to collect Young’s modulus, yield strength, and elongation. All the data were recorded for an averaged value of 5 specimens.

### 2.6. Rheological Test and X-ray Diffraction (XRD) of PBABI Copolyesters

Physica MCR-301 (Anton Paar, Graz, Austria) was performed to investigate the rheological tests using a cone plate type with a diameter of 25 mm with a gap of 1 mm at 80 °C. Dynamic strain sweep tests with 0.1% oscillatory strain and a set temperature step of 10 °C yielded the storage modulus, loss modulus, and complex viscosity at the angular frequency range of 0.1–100 rad s^−1^. In XRD analysis, the hot−melt mechanical compression with 80 °C and a pressure of 50 Kgf cm^−2^ for 1 min to prepare a thin film; Malvern Panalytical X’Pert 3 powder diffractometer (Malvern, UK) was adopted with CuKα radiation (λ = 0.154 nm) in 2θ from 10 to 40 degrees at RT with a scanning speed of 0.2° min^−1^ to collect the XRD patterns.

### 2.7. All-Atom Molecular Dynamics Simulation Procedures

All-atom simulation models were built with periodic boundary conditions (PBC), refs. [8,31] consisting of the EDTA, BTCA, and PE molecules as a center molecule formed four ester bonds with PBABI copolyesters, respectively, to investigate the role of different type modifiers for its mechanical property. The movement ability of the crosslinking point can be analyzed through density, mean square displacement (MSD), the radius of gyration (R_g_), and accessible surface area (ASA) analysis to investigate the structural deviation as a function of time. COMPASS force-field [32], which has shown robust performance in predicting structures and dynamics of organic molecules in all-atom molecular dynamics (AAMD) simulations, was performed for a simulation model to investigate the dynamic structural behavior. Isothermal–isobaric ensemble (NPT) at 1 atm and 298 K with a 1 fs time step were employed for all simulation models. Thermostat chosen was the Nose-Hoover method [33] (Q ratio of 0.01), barostat by Berendsen method (decay content of 0.1 ps), and the cutoff distance was selected in 15.5 Å. In the first step, the geometry minimization method was performed to relax and equilibrate the artificial models. After the equilibration procedure, the dynamic simulations were performed in 1000 ps with a time step of 1 fs at 298 K and 1 atm. The simulation results were checked to achieve the equilibrated state when the total energy deviation below 5%. In the analysis part, the AAMD simulation results were collected, and then the trajectory was evaluated to calculate deviations of density, R_g_, MSD, and ASA distribution as a function of simulation time.

## 3. Results and Discussion

**Figure 1:**
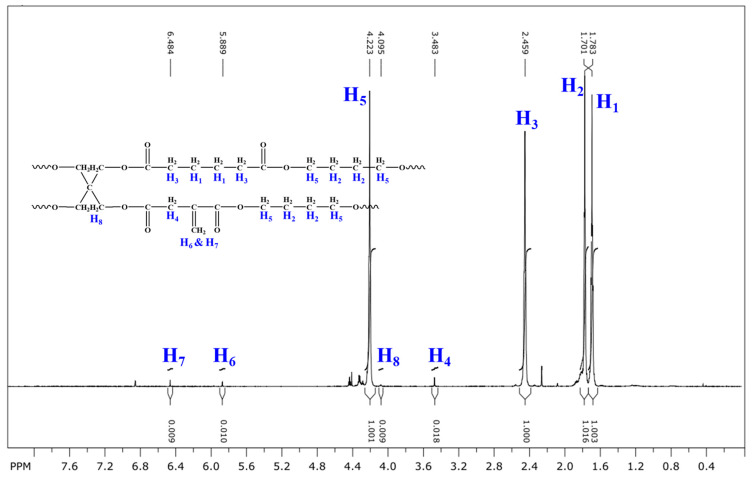
^1^H NMR spectra of PBABI copolyester with PE at a ratio of BA/BI = 9/1.

PBABI copolyester with PE at a proportion of BA/BI = 9/1 was validated by ^1^H NMR spectra and presented in Figure 1, as well as the other proportions of BA/BI illustrated in Figure S1. The feature peaks of PBABI copolyesters with various BA/BI rations were recognized and also assigned to H_1_ ranged from 1.700 to 1.712 ppm for 3,4-CH_2_ in adipic acid, H_2_ ranged from 1.960 to 1.794 ppm for 2,3-CH_2_ in 1.4-butanediol, H_3_ ranged from 2.459 to 2.477 ppm for 2,5-CH_2_ of adipic acid, H_4_ ranged from 3.483 to 3.501 ppm for 2-CH_2_ of itaconic acid, H_5_ ranged from 4.223 to 4.236 ppm for 1,4-CH_2_ of 1.4-butanediol, H_6_ ranged from 5.889 to 5.913 ppm for –C=CH_2_ of itaconic acid, H_7_ ranged from 6.484 to 6.501 ppm for –C=CH_2_ of itaconic acid, as well as H_8_ ranged from 4.089 to 4.107 ppm for –CH_2_ of the PE. Detailed NMR results in chemical shift, the integral ratio, and the calculated IA composition of PBABI copolyesters were recorded in Table S1. The calculated proportions of C=C bond of the IA were estimated in the values of 2.40%, 7.14%, 14.24%, 19.83%, and 28.79% for BA/BI = 9/1, 8/2, 7/3, 6/4, and 5/5, respectively, suggesting the C=C bonds of the IA may isomerize into monomethyl fumarate at high polymerization temperature [34]. Furthermore, near half content of the C=C bonds of IA can be reserved in various BA/BI rates, meaning about 50% C=C bonds may be created saturated C–C bonds to drive a partial crosslinking network in PBABI copolyesters [8,9]. Furthermore, Brännström et al. reported poly(butylene itaconate-*co*-butylene succinate) (PBIBSu) copolyesters, indicating the curing degree of the PBIBSu copolyesters with different BI/BSu ratios ranged near 50–75% [35]. As a result, keeping ~50% of the C=C bonds within PBABI copolyester was important to drive a higher partial network architecture to reduce the crystallinity and the backbone flexibility.

**Figure 2:**
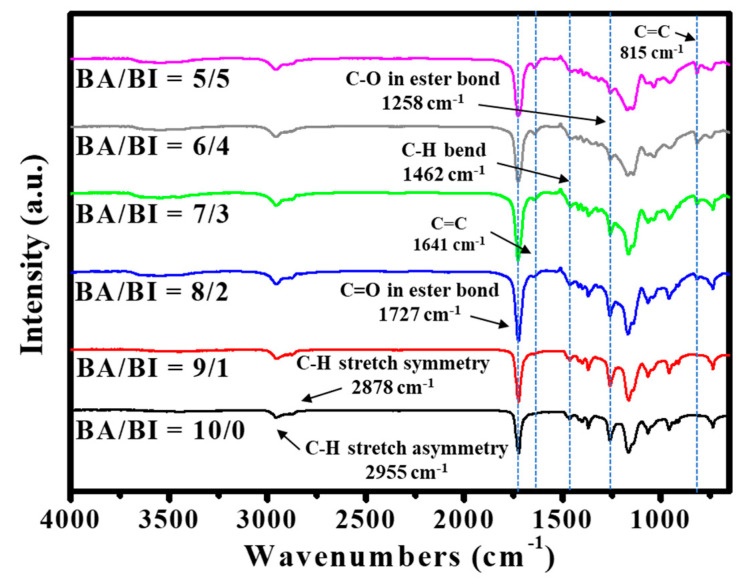
FT-IR spectra of PBABI copolyesters with different BA/BI ratios.

The feature absorption peaks of FT-IR spectra of the PBABI copolyesters display in Figure 2, for which the absorption peaks correlated to the asymmetry and symmetry C–H stretch has been found at 2955 and 2878 cm^−1^, respectively. Stretching vibration of the C=O in the ester bond at 1727 cm^−1^, a C–H bend absorption peak at a value of 1461 cm^−1^, and a band of 1258 cm^−1^ was related to C–O of the ester bond. The most significant peak was discovered and remarked in 1641 and 815 cm^−1^, which was corresponding to the C=C bond stretching vibration within IA. The intensity of these two absorption peaks can be improved by rasing the BI concentration, explaining that the IA molecule was effectively copolymerized into a main-chain of PBABI copolyesters. Furthermore, the C=C bond of IA can be preserved by 4-methoxyphenol, even through a high melt polymerization temperature above 230 °C; these preserved C=C bonds can be driven via the UV curing technique, to enhance the mechanical behavior of the PBABI copolyesters [8,9]. Tang and colleagues have observed that IA-based copolyesters can create a partially crosslinked architecture to tune the mechanical property by way of the UV curing strategies [36].

**Figure 3:**
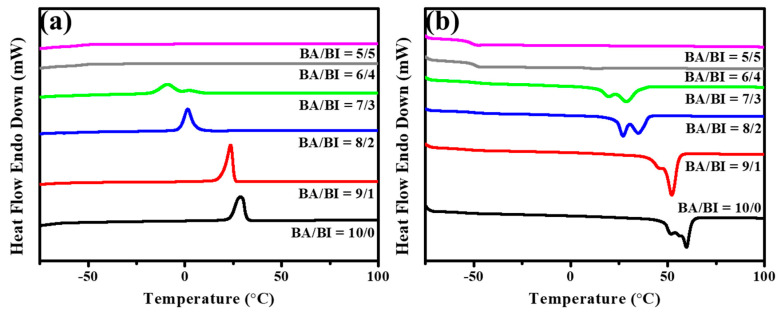
DSC trace of PBABI copolyesters with different BA/BI ratios: (**a**) cooling curves in the first scan, and (**b**) heating curves in the second scan.

Figure 3 displays the DSC trace of PBABI copolyesters. The cooling curves of the first cycle and heating curve of the second cycle exhibit in Figure 3a,b, respectively, indicated the crystallization occurred during the cooling procedures, and a single peak observed in the subsequent heating at a rate of 10 °C min^−1^. T_m_ values reduced from 59.5 to 19.5 °C as BI concentration increased to 30 mol%. Moreover, after removing the thermal history, the melting peak of the PBABI copolyesters was shown to be in a continuous double peak, implying that a competitive effect might exist in the crystallization zone of BA and BI due to different reactivity at the same melt polymerization temperature. The ∆H_m_ decreased from 42.3 to 27.1 J g^−1^ as BI unit increased, indicating that the crystallization region can be disrupted in the existence of the BI unit. Moreover, A single crystallization peak was noticed clearly around 2.3–28.2 °C at different BA/BI ratios during the cooling process, suggesting that the molecular chain of PBABI copolyester was easy to pack into the ordered phase. The IV values of PBABI copolyesters stayed at 0.68, 0.61, 0.62, 0.57, 0.52, and 0.38 dL g^−1^, with gradually increasing the BI content by 10 mol%, suggesting chain entanglement can be hindered by raising the BI concentrations to reduce the IV value. All the thermal properties and intrinsic viscosity are listed in Table 1.

**Figure 4:**
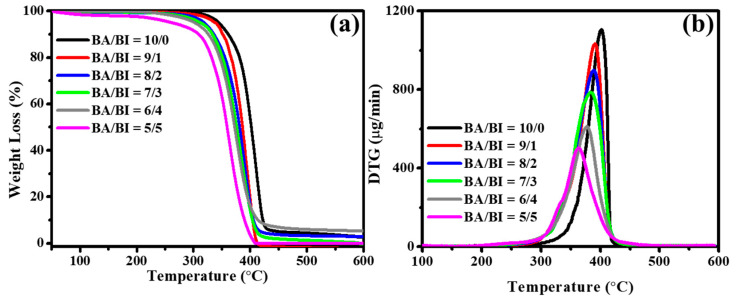
(**a**) Thermal gravimetric analyzer (TGA) and (**b**) derivative thermogravimetry (DTG) profiles of PBABI copolyesters with different BA/BI ratios.

The results of thermal gravimetric analysis (TGA) and derivative thermogravimetry (DTG) profiles of PBABI copolyesters are presented in Figure 4. In TGA analysis, the 5 wt% weight loss temperature (T_d-5%_) observed around 261.1–344.7 °C, representing PBABI copolyesters exhibit excellent thermal stability since the T_d-5%_ was over 300 °C, except for BA/BI = 5/5, the exact values can be seen in Table 1. Otherwise, PBABI copolyesters tend to amorphous polymer when the BI unit above 40 mol% and the thermal stability of PBABI copolyesters could be diminished with an increase of BI concentration. From DTG analysis (see Figure 4b), a single peak was observed, suggesting the PBABI copolymers with PE exhibited one-stage degradation and similar behavior in different BA/BI ratios, and the degradation behavior tends to shift to lower temperature as the BI unit increased.

**Figure 5:**
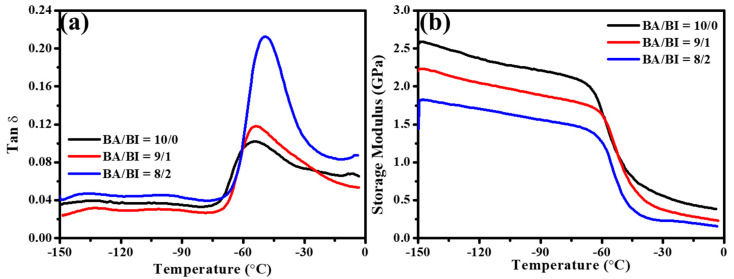
Dynamic mechanical analyzer results for (**a**) Tan δ and (**b**) storage modulus of PBABI copolyesters with a ratio from 10/0 to 8/2.

Figure 5 displays dynamic mechanical analyzer (DMA) results of the PBABI copolyesters with a ratio from 10/0 to 8/2. The PBABI copolyesters exhibit a durable viscose and fluidity property at RT as the BI unit above 30 mol%; hence, the sample cannot be shaped at RT to perform the DMA analysis. Glass transition temperature (T_g_) obtained from the peak of tan *δ*ranged from −48.1 and −54.6 °C in the presence of BI unit below 20 mol%, as shown in Figure 5a, indicating that the T_g_ values were not related to the BI concentrations in the existence of PE. Hence, PE may play a key role in sustaining the stability in the amorphous zone at a relatively low temperature around −50 °C, even when the BI ratio was higher, due to the double-bond of IA, which can maintain the amorphous regime. The β- and γ-relaxation was also monitored near −100 °C for the movement in the R–C=CH_2_ of IA and −130 °C for the motion of the side chain of BI unit. As can be seen in Figure 5b, the storage modulus displayed a higher values range of 1.6–2.6 GPa at −150 °C, suggesting an excellent mechanical and stability property in extremely low-temperature circumstances in the presence of PE within PBABI copolyesters. Moreover, when the temperature was above T_g_ in the rubbery state, storage modulus decreased with an increasing BI content, implying the double-bond of IA molecule could be stimulated to interrupt and destruction the geometrical regularity of the molecular chain in the amorphous regime.

**Figure 6:**
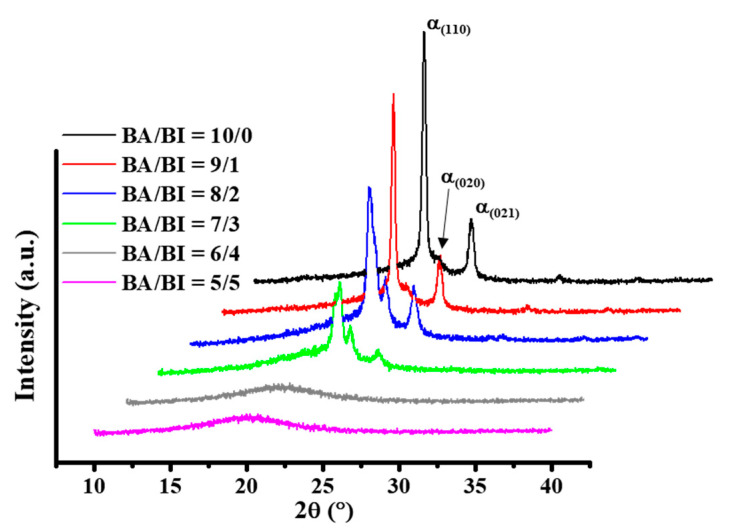
XRD patterns of PBABI copolyesters with different BA/BI ratios.

XRD patterns of PBABI copolyesters with varying BA/BI proportions in a 2θ range of 10–40°, as illustrated in Figure 6. XRD peaks in all BA/BI ratios were taken in 2θ values of 21.13°, 22.29°, and 24.24° for the crystal lattices of (110), (020), and (021), which were associated with the α-phase of PBA [37,38,39,40,41,42]. Moreover, XRD results of PBABI copolyesters in different BA/BI ratios have similar patterns compared to PBA, suggesting that the crystal panel of the BA unit could not be flustered by the existence of the BI unit below BA/BI = 7/3 and tend to amorphous state at BA/BI = 6/4 and 5/5. Otherwise, crystallinity values observed in 32.4%, 31.6%, 25.2%, and 21.4% for BA/BI = 10/0, 9/1, 8/2, and 7/3, respectively, indicated sufficient BI content could interrupt the chain packing into the ordered region to lowering crystallinity of PBABI copolyesters., which specified that the crystal formation was driven by the intermolecular van der Waals (vdW) interactions, and the PE can provide a nucleation site by a slight networking architecture formed. That is to say, PBABI copolyesters display an increase of chain flexibility with increasing BI concentration, which might have improved a more-effective rearrangement of the polymer chain, allowing vdW interactions to reduce the formation of the crystalline domain, and decreased the crystallinity of PBABI copolyesters. As a result, the determined crystallinities obtained via XRD results agree well with DSC measurement.

**Figure 7:**
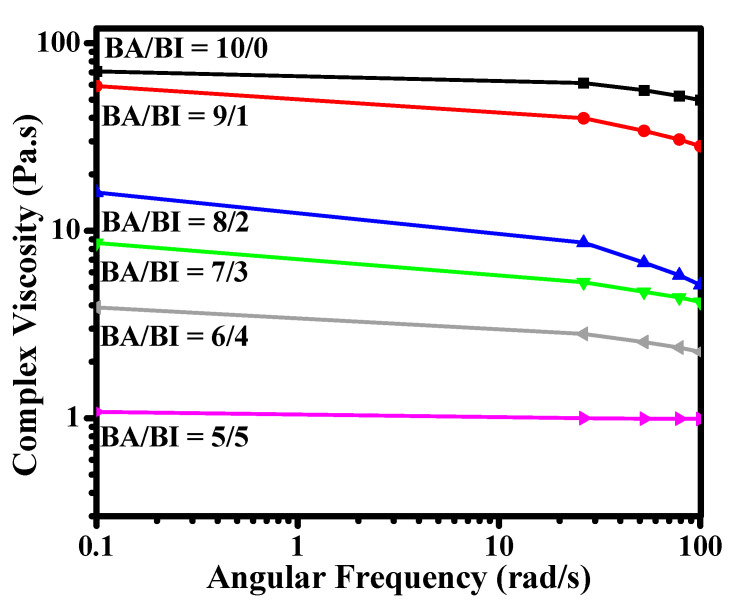
Complex viscosity of PBABI copolyesters with different BA/BI ratios as a function of angular frequency with 0.1–100 rad s^−1^ at 80 °C.

The complex viscosity (η) as a function of angular frequency using a cone-plate rheometer for various BA/BI ratios of PBABI copolyesters with 0.1 mol% PE at 80 °C was explored and displayed in Figure 7, examining the η was reduced gradually with increasing BI concentrations at an angular frequency range of 0.1–100 rad s^−1^. A shear-thinning behavior was obtained in BA/BI ratios from 10/0 to 6/4 around an angular frequency range of 20–30 rad s^−1^, suggesting PBABI copolyesters with PE at 80 °C represented a non-Newtonian fluid behavior along with the entire frequency range.

**Figure 8:**
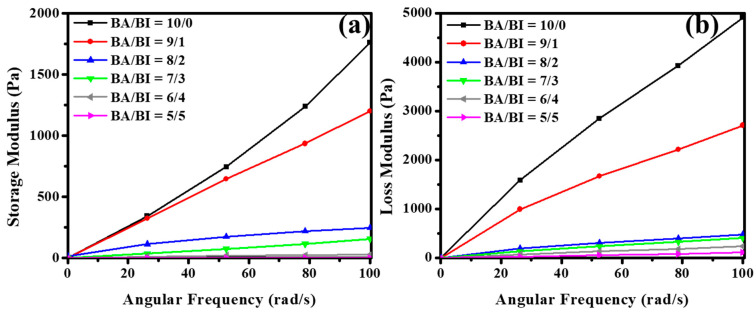
(**a**) Storage and (**b**) loss modulus of PBABI copolyesters with different BA/BI ratios as a function of angular frequency with 0.1–100 rad s^−1^ at 80 °C.

Figure 8a,b displays the storage and loss modulus curves of PBABI copolyesters as a function of frequency. Both storage and loss moduli gradually decreased with an increase of angular frequency, indicating molecular chain entanglement reduced in the presence of the BI unit. Furthermore, the storage modulus is associated with the melt elasticity of the polymer, and PBABI copolyester with PE tends to the viscose flow property dominates. Moreover, the loss modulus was exhibited higher values than the storage modulus at the entire angular frequency, suggesting the PBABI copolyesters preferred to characterize a viscose property, and the flexibility of PBABI copolyesters improved by BI units, leading to drive the fluidity and processability.

**Figure 9:**
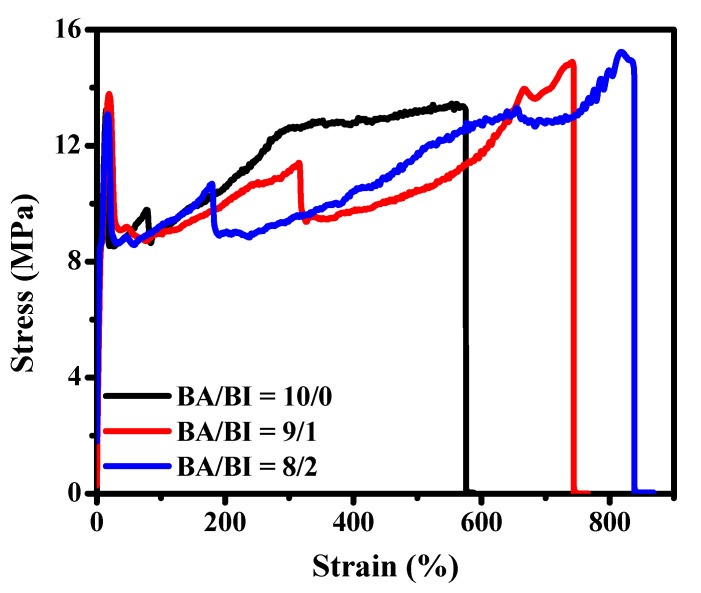
Stress-strain curves of PBABI copolyesters with a ratio from 10/0 to 8/2.

Stress-strain curve was taken via the tensile tests, as shown in Figure 9, signifying the macroscopic deformation of BA/BI = 100/0 was established at strength and strain of 13.2 MPa and 575.2%, respectively. Also, the strain behavior displays a soft and tough characteristic in BA/BI ratios from 10/0 to 8/2. The yield strength, elongation, and Young’s modulus at different BA/BI ratios were evaluated in a range of 13.2–13.8 MPa, 575.2–838.5%, and 65.1–83.8 MPa, respectively, suggesting the BI unit can act on behalf of a role in tuning the mechanical properties of PBABI copolymers. Furthermore, T_m_ value was decreased below RT with the BA/BI ratio above 7/3, implying the sample tends to represent a viscose feature to restrict the tensile test. 

Compared to our previous PBABI copolyesters with EDTA [8] and BTCA [9] as a modifier for its stress-strain curves (see Figure S2), a better tough behavior was found in PBABI copolyesters with PE as a modifier in the same BI ratio. However, a relatively rigid property was achieved in BTCA than PE as a modifier at the same BI ratios. Furthermore, this is not surprising that the BTCA within PBABI copolyesters has a little stiffer characteristic than EDTA due to a benzene ring in the center of BTCA. In brief, a slight content of modifier in 0.1 mol% within PBABI copolyesters can efficiently regulate the mechanical property, but the reason is unclear; therefore, we have constructed the artificial models with different types of modifiers to form networking architecture, to investigate correlation to the modifiers and mechanical behaviors by means of AAMD techniques.

**Figure 10:**
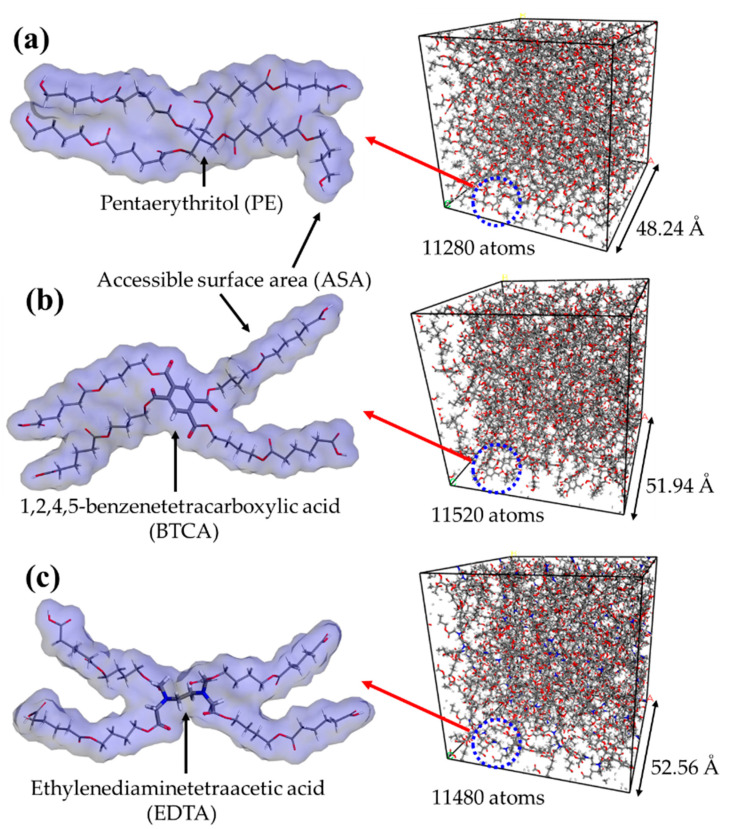
Snapshot of PBABI copolyester with (**a**) PE, (**b**) BTCA, and (**c**) EDTA as a modifier. Left: the molecular chain conformation, the modifier in the center of PBABI copolyesters. Right: the PBABI models with different modifiers. (Color: H, C, N, and O are presented in white, grey, blue, and red, respectively.) the accessible surface area is shown in a light blue color.

The models of PBABI copolyesters with different modifiers are presented in Figure 10, showing a different stereo conformation (Figure 10a PE in tetrahedral (flexible), Figure 10b BTCA in coplanar (rigid), and Figure 10c EDTA in tetrahedral (semi-rigid)). Cubic box size was determined in a length of 48.24, 51.94, and 52.56 Å with 11,280, 11,520, and 11,480 atoms for PE, BTCA, and EDTA as a modifier to build a networking structure, respectively. The snapshot of molecular chain conformation was taken after 1000 ps AAMD simulations, and the accessible surface area (represent in transparent light blue) was calculated from the vdW radius of 1.4 Å to examine the exposed surface area as a function of time.

**Figure 11:**
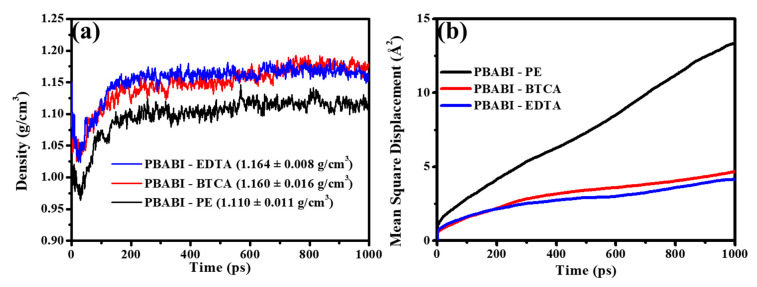
(**a**) Density and (**b**) mean square displacement profiles as a function of simulation time for PBABI copolyester with different modifiers of PE, BTCA, and EDTA.

Figure 11a shows the density evaluation as a function of time, observing PBABI with PE has the lowest density than BTCA and EDTA, implying the structure is more loose and flexible to obtain larger free volume, which could be beneficial to observe better elongation compared to BTCA and EDTA. Otherwise, EDTA exhibits a tetrahedral conformation that can drive the PBABI copolyester to achieve a stable packing to improve the density compared to BTCA. Moreover, the benzene structure in the center of BTCA represents a coplanar conformation, which could restrict the movement of the molecular chain to hinder the PBABI copolyester from reaching a relatively stable networking architecture [43]. Moreover, the mean square displacement (MSD) profile displays in Figure 11b, indicating the PBABI with PE has the most significant diffusion coefficient of 1.98 × 10^−3^ Å^2^ ps^−1^ than with both BTCA (5.5 × 10^−4^ Å^2^ ps^−1^) and EDTA (4.5 × 10^−4^ Å^2^ ps^−1^) due to a lower density, which reflected a more amorphous regime to promote the motion of molecular chain to enhance the elongation behavior to attain a better tough property in PBABI copolyesters. Otherwise, PBABI with BTCA has a larger MSD value than with EDTA, demonstrating the EDTA is a semirigid molecule, and is easy to obtain a compact packing of PBABI copolyesters to acquire the largest density and to restrict the molecular chain motion to gain a lower diffusion coefficient.

**Figure 12:**
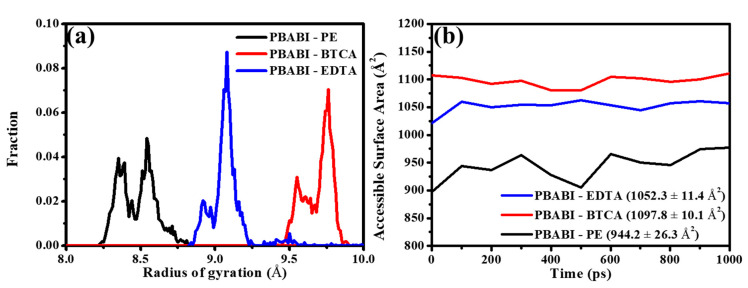
(**a**) Radius of gyration distributions and (**b**) surface area profile as a function of simulation time, for PBABI copolyester with different modifiers of PE, BTCA, and EDTA.

Figure 12a shows the radius of gyration (R_g_) distributions for PBABI copolyesters with different modifiers of PE, BTCA, and EDTA, displaying the lowest R_g_ value was located in PBABI copolyester with PE, which has a higher crosslinking density under the same modifier ratio and unit volume, implying the PE can restrict the movement of molecular chains to form a tighter network to exhibit a higher strain behavior. Thus, PBABI copolyester with BTCA has a larger R_g_ value than with EDTA, caused by the coplanar conformation of BCTA molecule to obstruct the molecular chain packing. The EDTA exhibits a semirigid property to improve the chain movement to obtain lower R_g_ values. Hence, PBABI copolyester with EDTA has a better strain characteristic than with BTCA. Furthermore, the benzene ring of BTCA can reflect a rigid characteristic to observe better stress and weaker strain performance, and EDTA represents a semirigid feature to a better strain behavior due to a better molecular chain packing, but lower than with PE. Figure 12b displays the accessible surface area (ASA) as a function of time, indicating the PBABI copolyester with PE has the smallest ASA value under the same modifier ratio and unit volume, implying a better compact stereo structure to achieve a better strain performance. The largest ASA value is located in PBABI copolyester with BTCA, reflecting a larger occupied volume that can drive better stress owing to the benzene structure. Hence, we observed that the tough property is associated with stereo architecture and molecular structure. In brief, a relatively high degree of crosslinking in unit volume and more flexibility of molecular chains can acquire a better strain property in the PBABI copolyesters.

## 4. Conclusions

A series of PBABI copolyesters with PE as a modifier was systematically produced through bulk polymerization strategies and identified via both NMR and FT-IR spectrum to explore the thermal, mechanical, and rheological properties. The values of T_m_ and T_c_ are reduced from 59.5 to 19.5 °C and 28.2 to −9.1 °C, respectively, as the BI unit increased to 30 mol%. T_g_ was located in a range of −54.6 to −48.1 °C and was influenced slightly by the BI content. IV values were dropped in gradually increasing the BI content, suggesting chain entanglement can be interrupted by raising BI concentrations to reduce the IV value. XRD results of PBABI copolyesters in different BA/BI ratios have similar patterns compared to PBA and transformed into the amorphous state by the existence of the BI unit above 40 mol%. The yield strength, elongation, and Young’s modulus in different BA/BI ratios were evaluated in a range of 13.2–13.8 MPa, 575.2–838.5%, and 65.1–83.8 MPa, respectively. Shear-thinning behavior was achieved in PBABI copolyesters with BA/BI ratios from 10/0 to 6/4 around an angular frequency range of 20–30 rad s^−1^. PBABI copolyester with PE has the most significant diffusion coefficient of 1.98 × 10^−3^ Å^2^ ps^−1^ due to lower density, which reflected a more amorphous regime to promote the motion of molecular chains to enhance the elongation behavior to attain a better tough property. A relatively high crosslinking degree in unit volume and more flexibility of molecular chains can develop a better strain performance to produce a tighter and well-dispersed stereo networking architecture in the PBABI copolyesters.

## Figures and Tables

**Table 1:** Thermal property and an intrinsic viscosity of PBABI copolyesters at different BA/BI ratios.

Sample	IV (dL g^−1^)	T_g_ (°C)	T_c_ (°C)	ΔH_c_ (J g^−1^)	T_m_ (°C)	ΔH_m_ (J g^−1^)	T_d−5%_ (°C)
BA/BI = 10/0	0.68	−54.6	28.2	−48.3	51.5, 59.5	42.3	344.7
BA/BI = 9/1	0.61	−53.3	23.6	−50.2	45.5, 52.4	41.3	338.0
BA/BI = 8/2	0.62	−48.1	1.3	−35.6	26.9, 34.8	33.2	318.7
BA/BI = 7/3	0.57	−50.1 (DSC)	−9.1	−30.8	19.5, 28.4	27.1	312.6
BA/BI = 6/4	0.52	−50.3 (DSC)	n/a	n/a	n/a	n/a	301.9
BA/BI = 5/5	0.38	−52.9 (DSC)	n/a	n/a	n/a	n/a	261.1

## Supplementary Materials

The following are available online at https://www.mdpi.com/2073-4360/12/9/2006/s1, Figure S1: ^1^H NMR spectra of PBABI copolyester with PE at a ratio of BA/BI = 10/0, 8/2, 7/3, 6/4, and 5/5, Figure S2: The stress-strain curves of PBABI copolyesters with (a) EDTA [8] and (b) BTCA [9] in different BA/BI ratios, Table S1: The composition of the calculated C=C bond of itaconic acid, PE ratio, chemical shifts (in ppm), and the integral ratio (value in brackets) of ^1^H NMR spectra for PBABI copolyesters with different BA/BI ratios.
