# Supplementary material for: Synthesis of Bio-Based Poly(Butylene Adipate-co-Butylene Itaconate) Copolyesters with Pentaerythritol: A Thermal, Mechanical, Rheological, and Molecular Dynamics Simulation Study
Source: Polymers (Basel). 2020 Sep 3;12(9):2006. doi: 10.3390/polym12092006 (PMC7564154; doi:10.3390/polym12092006)
Supplement: Supplementary file 1 [file polymers-12-02006-s001.pdf]

Supporting Information

Table S1. The composition of the calculated C=C bond of itaconic acid, PE ratio, chemical shifts (in ppm), and the integral ratio (value in brackets) of ^{1}H NMR spectra for PBABI copolyesters with different BA/BI ratios.

**Sample**	**Calculated IA ratio**	**Calculated PE ratio**	**H_1**	**H_2**	**H_3**	**H_4**	**H_5**	**H_6**	**H_7**	**H_8**
BA/BI = 10/0	0	0.17	1.701<br>(1.003)	1.785<br>(0.986)	2.463<br>(1.000)		4.226<br>(0.977)			4.096<br>(0.010)
BA/BI = 9/1	2.40	0.15	1.701<br>(1.003)	1.783<br>(1.016)	2.459<br>(1.000)	3.483<br>(0.018)	4.223<br>(1.001)	5.889<br>(0.010)	6.484<br>(0.009)	4.095<br>(0.009)
BA/BI = 8/2	7.14	0.21	1.700<br>(1.012)	1.783<br>(1.030)	2.465<br>(1.000)	3.489<br>(0.057)	4.224<br>(1.034)	5.895<br>(0.028)	6.489<br>(0.032)	4.089<br>(0.014)
BA/BI = 7/3	14.24	0.28	1.712<br>(1.002)	1.794<br>(1.017)	2.477<br>(1.000)	3.501<br>(0.124)	4.236<br>(1.098)	5.913<br>(0.068)	6.501<br>(0.064)	4.101<br>(0.020)
BA/BI = 6/4	19.83	0.31	1.703<br>(1.021)	1.784<br>(1.009)	2.464<br>(1.000)	3.486<br>(0.190)	4.226<br>(1.046)	5.898<br>(0.091)	6.492<br>(0.096)	4.107<br>(0.024)
BA/BI = 5/5	28.79	0.24	1.706<br>(1.011)	1.783<br>(0.956)	2.459<br>(1.000)	3.495<br>(0.307)	4.224<br>(1.207)	5.901<br>(0.158)	6.495<br>(0.160)	4.101<br>(0.021)

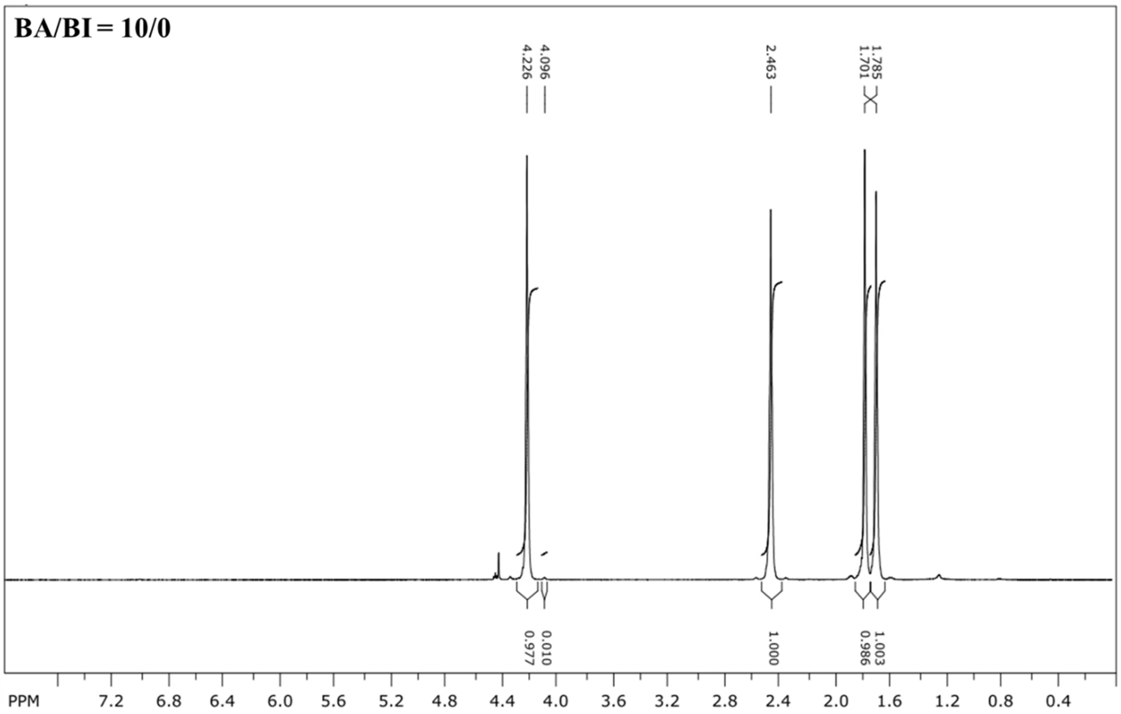
BA/BI = 10/0
4.226
4.096
2.463
1.785
1.701
0.977
0.010
1.000
0.986
1.003
PPM
7.2 6.8 6.4 6.0 5.6 5.2 4.8 4.4 4.0 3.6 3.2 2.8 2.4 2.0 1.6 1.2 0.8 0.4

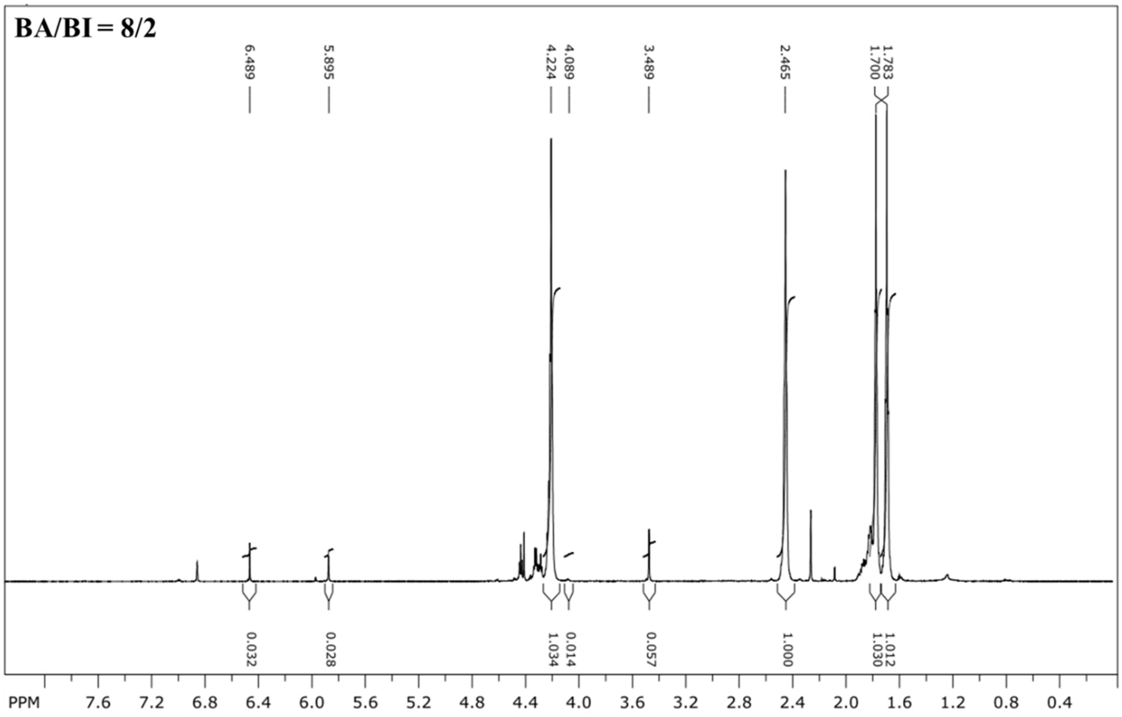
BA/BI = 8/2
6.489
5.895
4.224
4.089
3.489
2.465
1.783
1.700
0.032
0.028
1.034
0.014
0.057
1.000
1.030
1.012
PPM
7.6 7.2 6.8 6.4 6.0 5.6 5.2 4.8 4.4 4.0 3.6 3.2 2.8 2.4 2.0 1.6 1.2 0.8 0.4

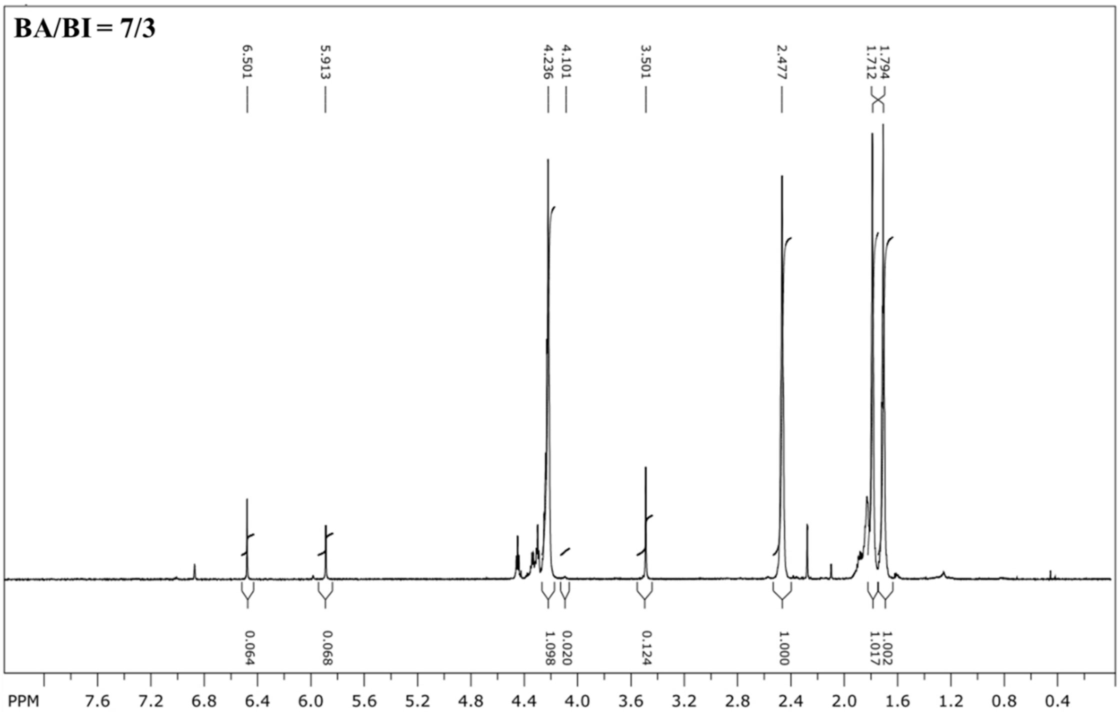
BA/BI = 7/3
6.501
5.913
4.236
4.101
3.501
2.477
1.794
1.712
0.064
0.068
1.098
0.020
0.124
1.000
1.017
1.002
PPM
7.6 7.2 6.8 6.4 6.0 5.6 5.2 4.8 4.4 4.0 3.6 3.2 2.8 2.4 2.0 1.6 1.2 0.8 0.4

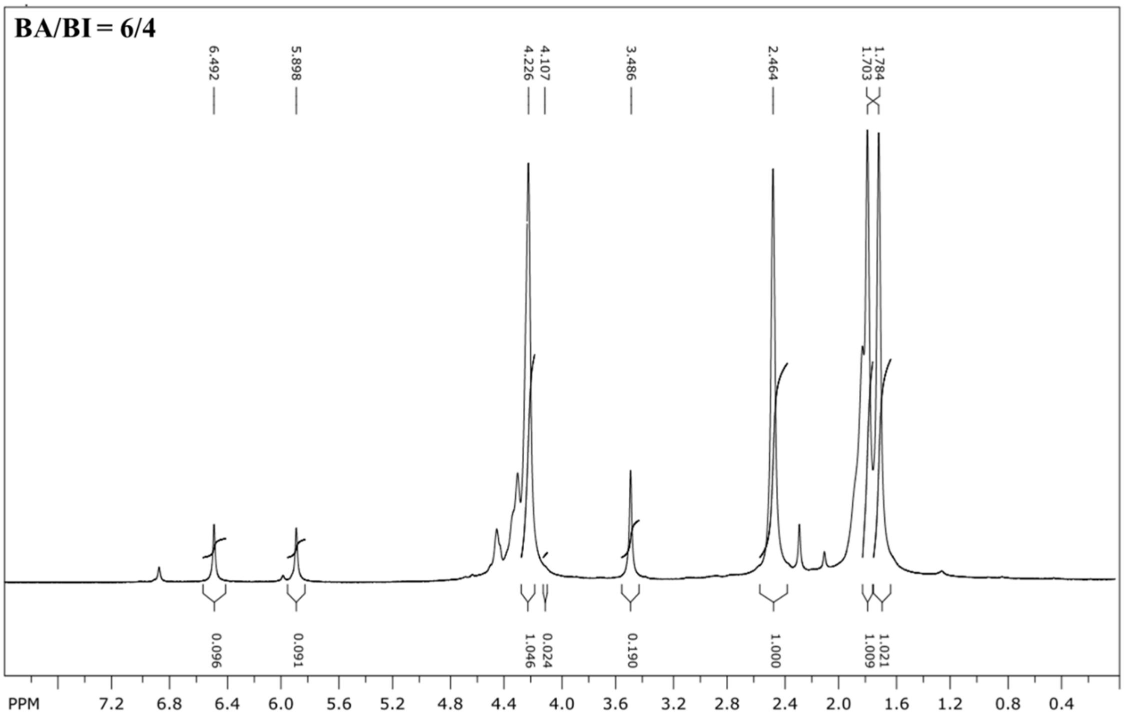
BA/BI = 6/4
6.492
5.898
4.226
4.107
3.486
2.464
1.784
1.703
0.096
0.091
1.046
0.024
0.190
1.000
1.009
1.021
PPM
7.2 6.8 6.4 6.0 5.6 5.2 4.8 4.4 4.0 3.6 3.2 2.8 2.4 2.0 1.6 1.2 0.8 0.4

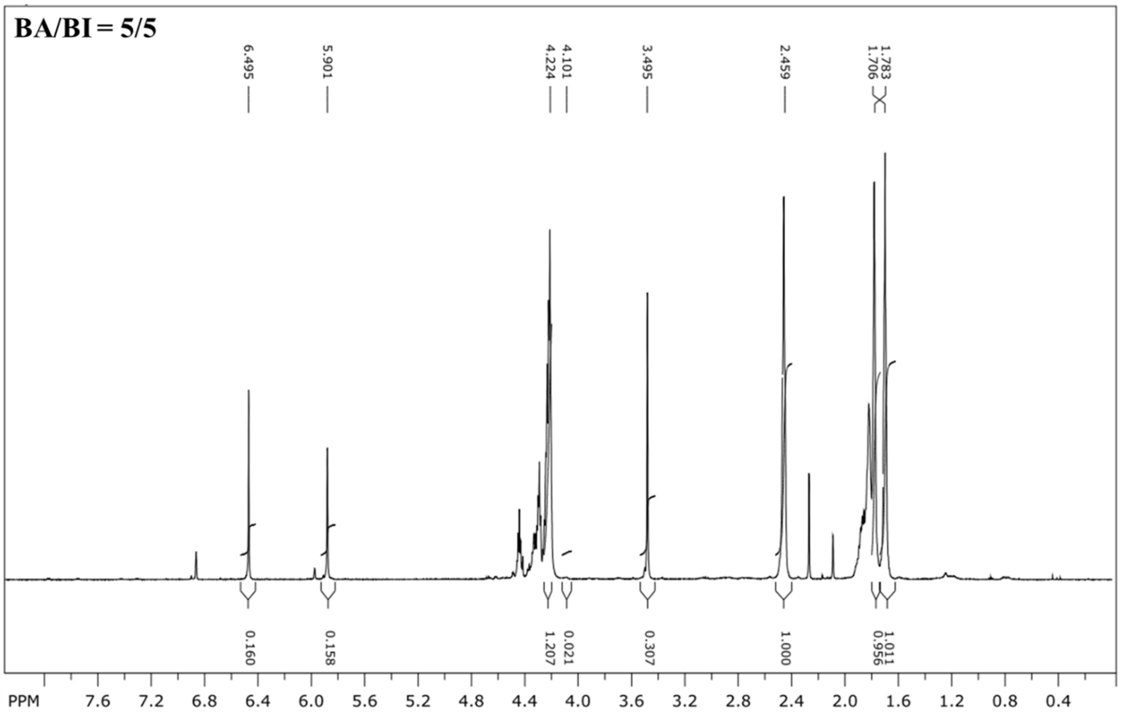

Figure S1. ^{1}H NMR spectra of PBABI copolyester with PE at a ratio of BA/BI = 10/0, 8/2, 7/3, 6/4, and 5/5.

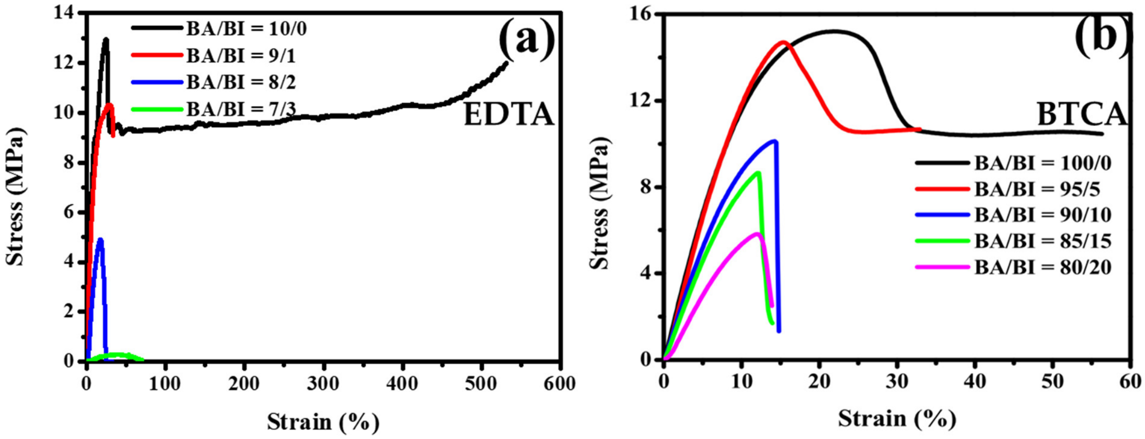

Figure S2. The stress-strain curves of PBABI copolyesters with (a) EDTA[8] and (b) BTCA[9] in different BA/BI ratios.
